# Game-changing restraint of Ros-damaged phenylalanine, upon tumor metastasis

**DOI:** 10.1038/s41419-017-0147-8

**Published:** 2018-02-02

**Authors:** Geraldine Gueron, Nicolás Anselmino, Paula Chiarella, Emiliano G. Ortiz, Sofia Lage Vickers, Alejandra V. Paez, Jimena Giudice, Mario D. Contin, Daiana Leonardi, Felipe Jaworski, Verónica Manzano, Ariel Strazza, Daniela R. Montagna, Estefania Labanca, Javier Cotignola, Norma D´Accorso, Anna Woloszynska-Read, Nora Navone, Roberto P. Meiss, Raúl Ruggiero, Elba Vazquez

**Affiliations:** 1 Departamento de Química Biológica, Universidad de Buenos Aires, Facultad de Ciencias Exactas y Naturales, Laboratorio de inflamación y Cáncer, Buenos Aires, Argentina; 2 CONICET- Universidad de Buenos Aires, Instituto de Química Biológica de la Facultad de Ciencias Exactas y Naturales (IQUIBICEN), Buenos Aires, Argentina; 3 Laboratory of Experimental Oncology, IMEX-CONICET (National Council of Scientific and Technical Research), National Academy of Medicine, Buenos Aires, Argentina; 4 Department of Cell Biology and Physiology, School of Medicine, University of North Carolina at Chapel Hill, Chapel Hill, NC USA; 5 Department of Organic Chemistry, CIHIDECAR-CONICET, FCEN, University of Buenos Aires, Buenos Aires, Argentina; 6 Department of Pharmacology and Therapeutics, Roswell Park Cancer Institute, Buffalo, NY USA; 7 Department of Genitourinary Medical Oncology, The University of Texas, M.D. Anderson Cancer Center, Houston, TX USA; 8 Laboratory of Pathology, Institute of Oncological studies (IEO) National Academy of Medicine, Buenos Aires, Argentina

## Abstract

An abrupt increase in metastatic growth as a consequence of the removal of primary tumors suggests that the concomitant resistance (CR) phenomenon might occur in human cancer. CR occurs in murine tumors and ROS-damaged phenylalanine, meta-tyrosine (m-Tyr), was proposed as the serum anti-tumor factor primarily responsible for CR. Herein, we demonstrate for the first time that CR happens in different experimental human solid tumors (prostate, lung anaplastic, and nasopharyngeal carcinoma). Moreover, m-Tyr was detected in the serum of mice bearing prostate cancer (PCa) xenografts. Primary tumor growth was inhibited in animals injected with m-Tyr. Further, the CR phenomenon was reversed when secondary implants were injected into mice with phenylalanine (Phe), a protective amino acid highly present in primary tumors. PCa cells exposed to m-Tyr in vitro showed reduced cell viability, downregulated NFκB/STAT3/Notch axis, and induced autophagy; effects reversed by Phe. Strikingly, m-Tyr administration also impaired both, spontaneous metastasis derived from murine mammary carcinomas (4T1, C7HI, and LMM3) and PCa experimental metastases. Altogether, our findings propose m-Tyr delivery as a novel approach to boost the therapeutic efficacy of the current treatment for metastasis preventing the escape from tumor dormancy.

## Introduction

Concomitant tumor resistance (CR) is the phenomenon in which a tumor-bearing host inhibits the growth of secondary tumor implants. Ehrlich^1^ first described it in 1906, but this phenomenon remained forgotten for about 60 years. After its renascence, it was demonstrated that both immunogenic and non-immunogenic tumors could induce CR in different animal models^2^. CR may be relevant to understand putative mechanisms of metastases control on the basis that metastases could be considered as secondary tumor implants developed spontaneously during the primary tumor growth^3^. Management of metastasis continues to be the Achiles’ heel of cancer^4^, since in many types of cancers, patients’ tumor relapse and often the responses produced to the adjuvant therapy are palliative and unpredictable.

Different explanations were proposed to address CR. The immunological hypothesis detailed how the growth of a tumor triggered an anti-tumor immune response, not strong enough to impair the growth of the primary tumor, but capable of suppressing the development of the secondary tumor inoculum^5^. However, the CR phenomenon was also observed in the absence of an immune reaction^6,7^. Non-immunological explanations included atrepsis^1^. However, others implied that the production and secretion of anti-proliferative or anti-angiogenic molecules by the primary tumor, limited the replication potency of tumor cells at secondary sites^6^.

In previous papers, using murine tumors widely different in origin, histology, and immunogenicity, we demonstrated that two temporally separate events of CR are detected during primary tumor growth^7,8^. The first event was only induced by small (≤500 mm^3^) immunogenic tumors, it was tumor-specific and thymus-dependent, and a typical immunological rejection was observed histologically at the site of the second tumor implant undergoing CR. The second event of CR was mediated by most large-sized (≥2000 mm^3^) immunogenic and non-immunogenic tumors and its intensity was proportional to tumor mass. In addition, the second event of CR was tumor-non-specific, thymus-independent, and it was unassociated with well-characterized growth-inhibitory molecules such as interferons, tumor necrosis factor-α, transforming growth factor (TGF)-β, angiostatin, and so on^6,8^, but with the serum factor(s) meta-tyrosine (*m-*Tyr) and ortho-tyrosine (*o*-Tyr)^9^, two isomers of tyrosine not present in normal proteins, *m-*Tyr and *o*-Tyr were responsible for 90% and 10%, respectively, of the total serum anti-tumor activity, as demonstrated by in vitro and in vivo experiments on the growth of three different murine tumors that induce CR and on a fourth tumor that does not induce CR, suggesting that *m-*Tyr can inhibit the proliferation of both types of tumors^10^. An excess of para-tyrosine (*p*-Tyr), the natural occurring isomer of tyrosine^10^, could not counteract the effect of *m*-Tyr and *o*-Tyr. In contrast, phenylalanine (Phe) reverted the inhibitory effects produced by both tyrosine isomers in a dose-dependent manner^10^.

In this work, we demonstrated for the first time that the phenomenon of CR may be induced by different experimental human solid tumors (prostate, lung anaplastic, and nasopharyngeal carcinoma) growing in immune-deficient nude mice and that the kinetics of this CR paralleled the second event of CR induced by murine tumors. We also show that *m-*Tyr mediates CR induced by human tumors (using the prostate PC3 line as model) and identify the signaling pathways involved. Moreover, we demonstrated that *m-*Tyr administration impairs spontaneous and experimental metastasis derived from murine and human tumors, mammary carcinomas (4T1, C7HI, and LMM3), and PCa experimental metastases, ascertaining it as a potential therapeutic factor for cancer metastasis.

## Results

### Concomitant resistance occurs in experimental human xenografts

**Fig. 1:**
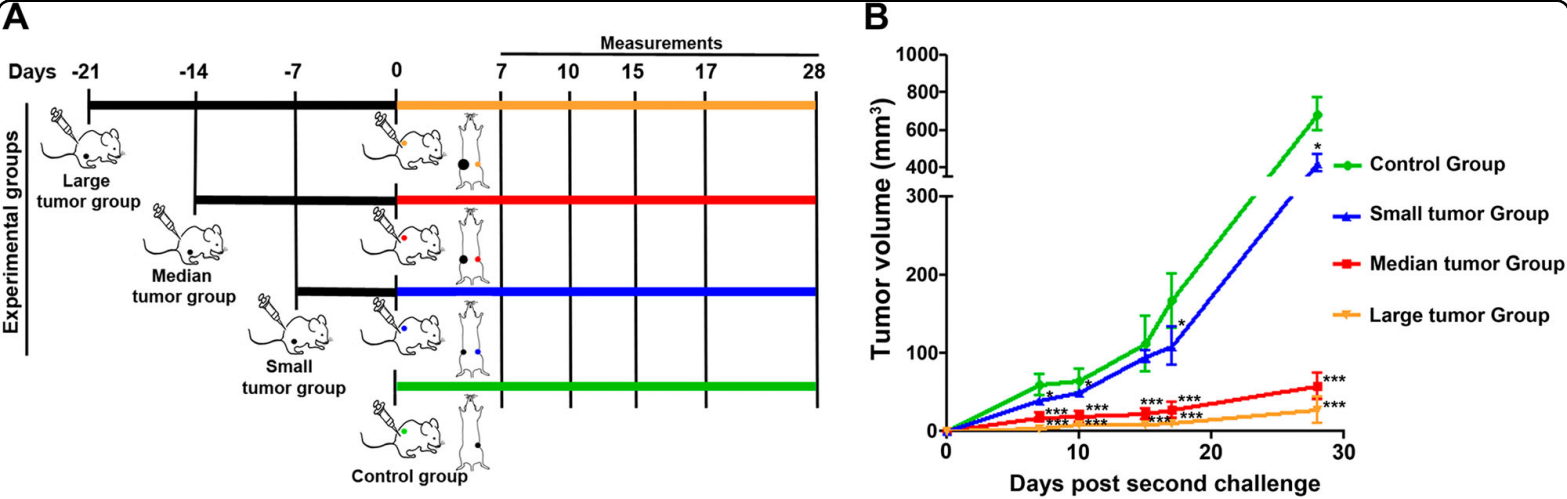
Concomitant resistance occurs in PCa **a** Schematic representation of CR strategy. **b** Male athymic *(nu/nu*) mice of 6–8 weeks old were randomized into four groups: control (*n* = 5) and three experimental groups (small, medium, and large primary tumors (*n* = 5)). Tumor cells (5 × 10^6^) were s.c. injected in the right flank at −21, −14, and −7 days of the experimental groups (primary tumors). At day 0, a second inoculation (3 × 10^6^ tumor cells) was performed in the left flank of the control and experimental groups (secondary tumors). Body weight and tumor growth were measured every 2 days starting at 7 days after inoculation when the tumors became detectable under the skin. One representative from at least three independent experiments is shown. The graph shows the average tumor volumes ± S.E.M. *P < 0.05, ***P < 0.001

To determine whether CR was occurring in human xenografts, we analyzed different solid tumor experimental models. Male *nu/nu* mice of 8–10 weeks old were randomized into two groups. Human PCa cells were injected s.c. in the right flank of the experimental group (primary tumor-bearing mice) and, at selected times (7, 14, or 21 days) after tumor inoculation—when PC tumor volumes were 101 ± 17, 317 ± 42, or 752 ± 114 mm^3^ (mean ± S.E.M.), respectively—a secondary tumor implant was carried out in the left flank. Control mice only received the tumor implant in the left flank (Fig. 1a). Body weight and tumor growth were measured every 2 days starting at 8 days after inoculation when tumors became palpable under the skin. The growth of the secondary tumor implants was significantly inhibited in the experimental group and the intensity of this inhibition was proportional to the primary tumor volume at the time of the secondary tumor implant: the larger the primary tumor volume, the stronger the inhibition of the secondary tumor implant (Fig. 1b).

We extended our findings and confirmed the CR phenomenon in two other experimental human solid tumor models, Calu6 (lung anaplastic carcinoma)^11^ and KB (nasopharyngeal carcinoma)^12,13^. As occurred with the PC model, Calu-6 and KB tumors clearly showed a significant reduction in the growth of the secondary implants that was proportional to the primary tumor volume at the time of the secondary tumor implant (Supplementary Fig. 1).

### Anti-proliferative activity of serum from mice bearing subcutaneous prostate tumors

**Fig. 2:**
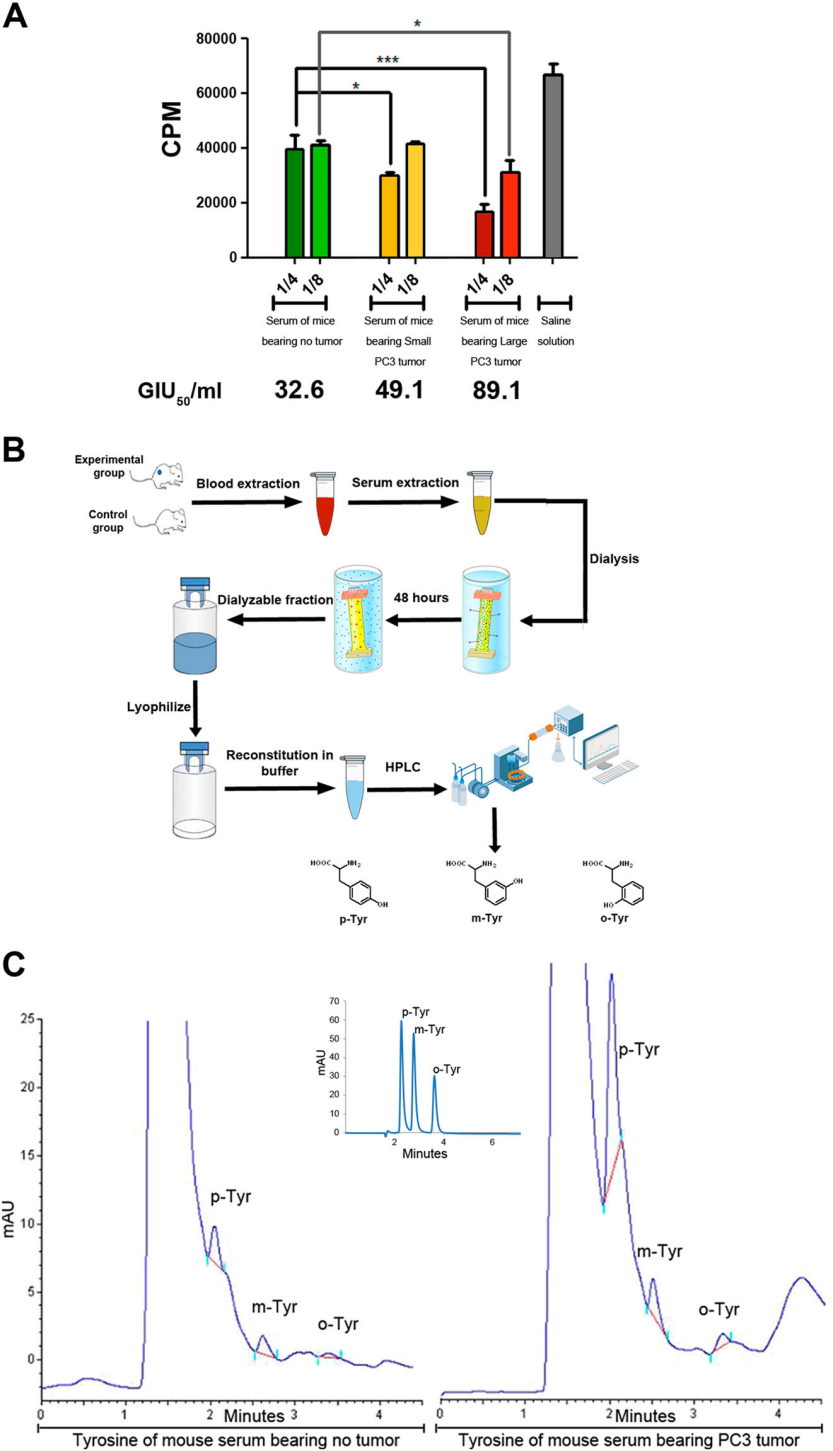
Anti-proliferative activity of serum from mice bearing subcutaneous prostate tumors **a** PC3 cells (3 × 10^5^) were exposed to twofold serum dilutions 1:4 and 1:8 (24 h), and 1 mCi/ml of [3H]-thymidine. Radioactivity incorporated into the cells was determined in a β-counter (Beckman). The titer of growth-inhibitory activity was defined as the reciprocal of the serum dilution producing 50% inhibition of [3H]-thymidine uptake by tumor cells as compared with [3H]-thymidine uptake by tumor cells cultured in medium only (GIU50 per ml). Results show the mean ± S.E.M of three samples of normal serum, three samples of serum from mice bearing large PC3 tumors (1700 mm^3^ approximately), and three samples of serum from mice bearing small PC3 tumors (500 mm^3^ approximately). Each dilution of each serum was assayed in triplicate. *P < 0.05; ***P < 0.001 significant difference. **b** Strategy of *p*-tyrosine (*p*-Tyr), *m-Tyr*osine (*m*- Tyr), and *o*-tyrosine (*o*-Tyr) purification from PC3 tumor-bearing and control mice serum. **c** *p*-tyrosine (*p*-Tyr), *m-Tyr*osine (*m*- Tyr), and *o*-tyrosine (*o*-Tyr) determinations were carried out using an HPLC-UV methodology previously developed (Ruggiero et al.^10^). Briefly the separation of *p*-Tyr, m-Tyr, and *o*-Tyr was performed in a C-18 Zorbax column (Agilent, 7.5 mm × 4.6 mm i.d., 5 μm particle size). The mobile phase consisted in a mixture of water:methanol:trifluoroacetic (87:12:1), the flow rate was set at 0.5 ml per min and the UV detection was performed at 280 nm. The right panel shows the retention times for *p*-Tyr, *m*-Tyr, and *o*-Tyr standards used for validation. One representative from at least three independent experiments is shown. SS saline solution

To test whether the serum from mice bearing subcutaneous prostate tumors had anti-tumoral activity, PC3 cultures were exposed to twofold serum dilutions 1:4 and 1:8 for 18 h, and [^3^H]-thymidine incorporation was measured. The titer of the growth-inhibitory activity was defined as the reciprocal of the serum dilution producing 50% inhibition of [^3^H]-thymidine uptake by tumor cells as compared to controls (GIU_50_ per ml). Serum was obtained from animals bearing either a large tumor (1875 mm^3^) or a small tumor (400 mm^3^). Results showed a significant increase in the GIU_50_ per ml for both sera compared with controls, with significantly higher anti-proliferative activity for serum from mice bearing large prostate tumors (Fig. 2a). Accordingly, serum from animals with small (100 mm^3^) and large Calu6 tumor (1700 mm^3^) presented an elevated GIU_50_ per ml compared to controls (Supplementary Fig. 2). These results suggested a direct correlation between the anti-tumor activity found in serum and the intensity of CR produced by each tumor at different stages of tumor growth.

### Identification of m-Tyr and o-Tyr in the serum of mice bearing PCa xenografts

Next, sera from mice bearing subcutaneous prostate tumors were purified and analyzed. *p*-Tyr, *m*-Tyr, and *o*-Tyr were identified by high-performance liquid chromatography (HPLC)-ultraviolet (UV) methodology^10^. The mobile phase consisted of a mixture of water:methanol:trifluoroacetic (87:12:1), the flow rate was set at 0.5 ml per min, and the UV detection was performed at 280 nm (Fig. 2b). Standards for *p*-Tyr, *m-Tyr*, and *o*-Tyr were used for validation (Fig. 2c). Results confirmed the presence *p*-Tyr, *m*-Tyr, and *o*-Tyr in control and experimental samples with a significant increase of the three metabolites in sera from mice bearing subcutaneous prostate tumors (Fig. 2c).

### m-Tyr treatment mimics concomitant resistance occurring in human xenografts

**Fig. 3:**
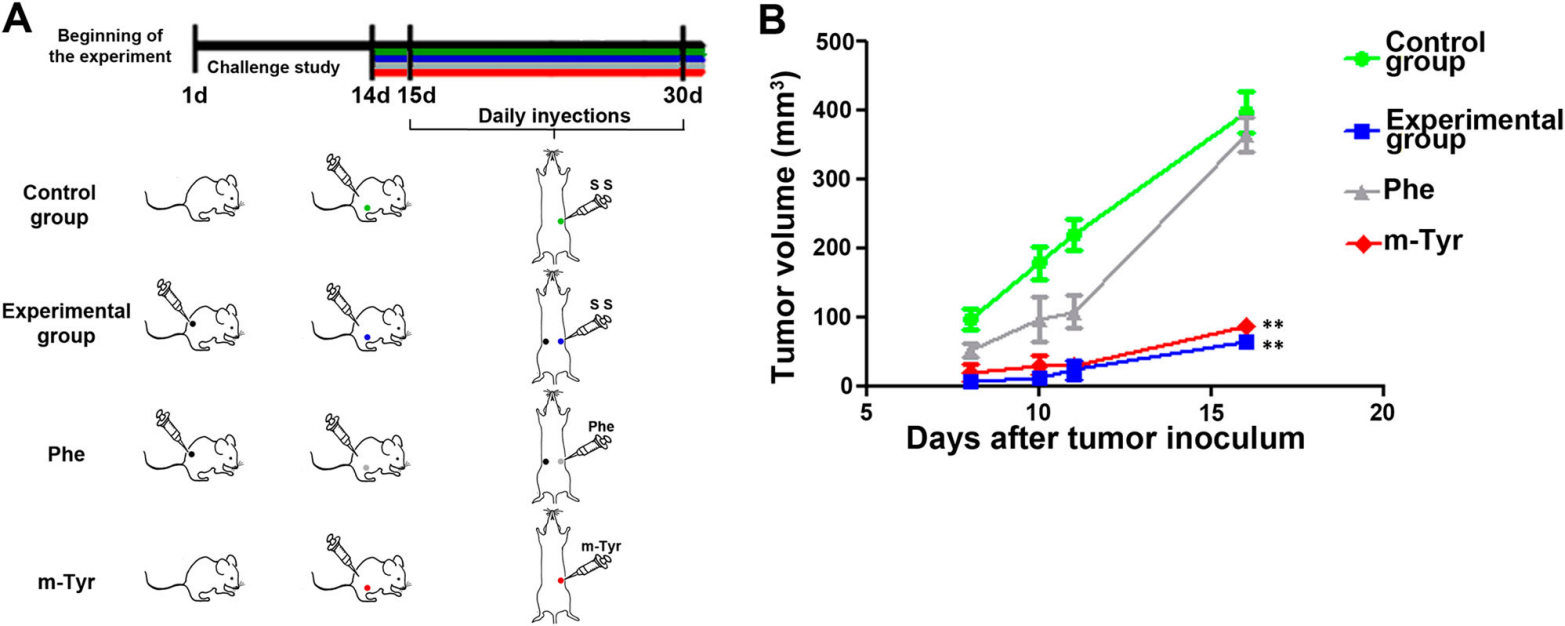
*m*-Tyr treatment mimics concomitant resistance in human xenografts **a** Counteracting effect of phenylalanine (Phe) on tumor growth inhibition induced by CR and inhibitory effect of *m*-Tyr on in vivo tumor growth mimicking the inhibition induced by CR. **b** The animals were randomly assigned into four groups: control; CR; Phe and *m*-Tyr. PC3 cells (5 × 10^6^) were s.c. inoculated in the right flank of animals of the CR and Phe groups. A total of 3 × 10^6^ cells were inoculated in the left flank of the animals of all groups 14 days after the first challenge. Both, Phe and *m*-Tyr groups were injected, in the left flank at the site of the tumor implant with Phe or *m*-Tyr (0.2 ml of 500 mg/ml, *n* = 10) respectively, for 15 days on a daily basis. Tumor growth was measured every 2 days starting 8 days after inoculation when the tumors were palpable. The graph shows the average tumor volumes of left flank implants ± S.E.M. One representative from at least five independent experiments is shown. **P < 0.01

Given that *m*-Tyr anti-tumor power proved to be 10 times more robust than that of *o*-Tyr^10^, we furthered our studies using this agent in PCa models. We assessed whether *m*-Tyr could mimic CR occurring in human PCa and that Phe could reverse this effect. Animals were randomly assigned into four groups: control; experimental (CR); Phe and *m*-Tyr. PC3 cells were s.c. inoculated (first challenge) in the right flank of animals of the CR and Phe groups. A second challenge was performed 14 days later in the left flank of the animals of all groups (Fig. 3a) and 24 h later, Phe and *m*-Tyr groups received the respective amino acids in the same site for 16 consecutive days (Fig. 3a). The RC phenomenon was recapitulated as the one observed in Fig. 1 (83.88% tumor volume decrease, **P < 0.01). *m*-Tyr group displayed significant tumor volume decrease compared to the control group by an average of 78.33% (**P < 0.01). Interestingly, Phe group presented a significant increase in tumor volume compared to the CR group (5.7 fold induction, **P < 0.01), further evidencing the counteraction of Phe on the CR phenomenon (Fig. 3b).

### m-Tyr treatment inhibits experimental and spontaneous metastasis

**Fig. 4:**
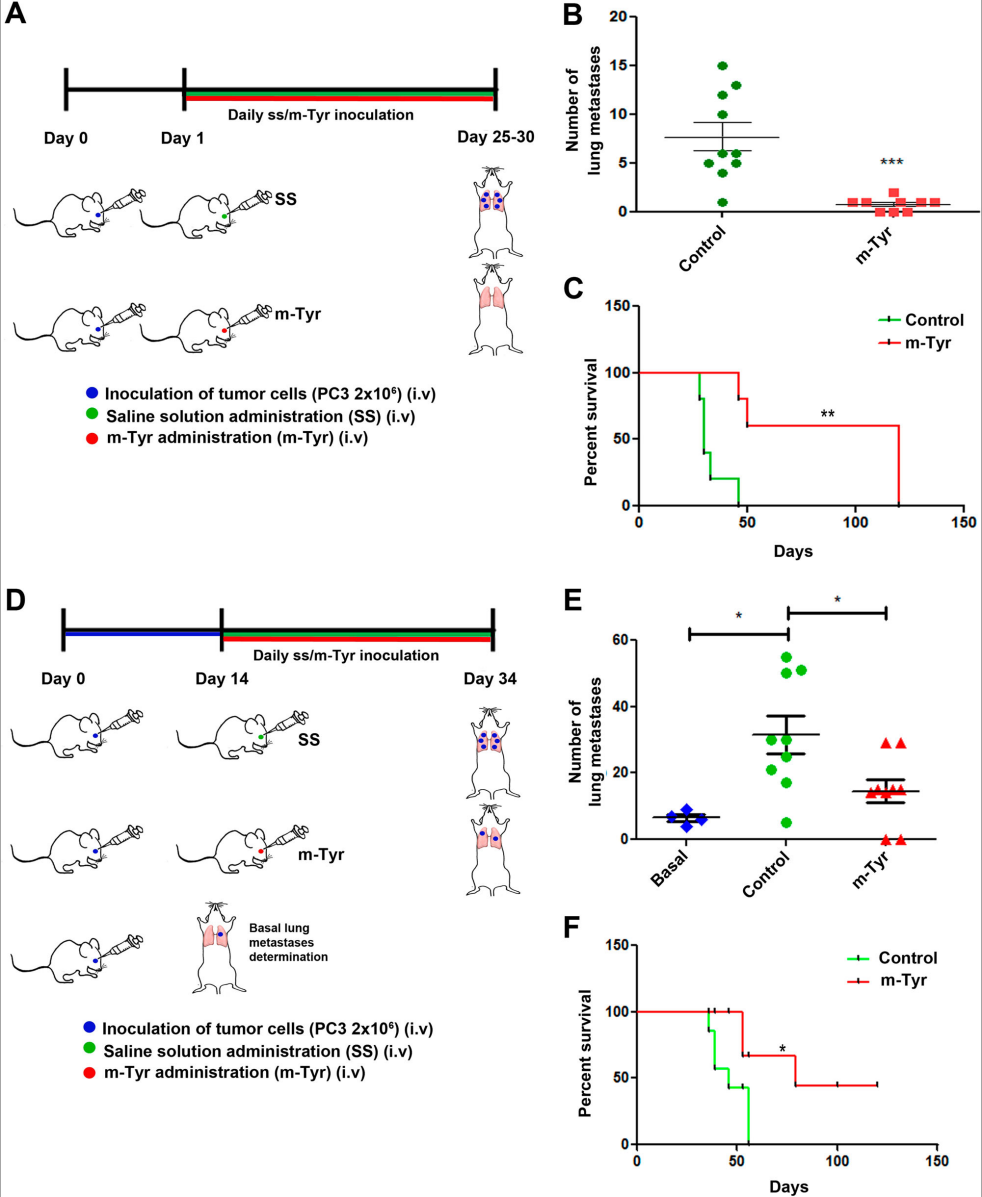
*m*-Tyr inhibits experimental metastasis of PCa **a**, **d** Schematic representation of the in vivo assay carried out to assess the role of *m*-Tyr in PCa experimental metastasis. Nude mice (*n* = 19) were i.v. challenged with 2 × 10^6^ PC3 tumor cells, and were subsequently injected with *m*-Tyr (*n* = 9) or saline (*n* = 10) on a daily basis (i.v. administration; 67 mg/kg/day). Animals were killed 20–25 days after tumor cell inoculation and evaluated for pulmonary metastases. **b** Pulmonary metastases were counted under a dissecting microscope. **c** Percent survival of nude mice (*n* = 10) inoculated with 2 × 10^6^ PC3 tumor cells i.v. (day 0). The figure shows the percentage of the survivors of *m*-Tyr-treated and control mice (saline solution i.v. administration) as a function of the days after tumoral cells injection. **d** Schematic representation of PCa experimental metastasis delivering *m*-Tyr 14 days post tumor cell inoculation, when metastases were already established in lung. **e** Pulmonary metastases were counted under a dissecting microscope. **f** Percentage of survivors of *m*-Tyr-treated and control mice (saline solution i.v. administration) as a function of the days after tumoral cells injection (2 × 10^6^ PC3 cells). *P < 0.05, **P < 0.01, *** *P* < 0.001 significant difference

Human tumors do not produce spontaneous metastases when growing into *nu/nu* mice. For this reason, *m*-Tyr effect was tested in a model of experimental PCa metastasis. Nude mice were i.v. challenged with PC3 tumor cells and starting 1 day later (when metastases were not yet established) or 14 days later (when metastases were already established in lung), mice were injected with *m*-Tyr (treated groups) or saline (control groups) on a daily basis for the following 21 days (Fig. 4a, d). At days 21 or 34 after tumor inoculation, animals were killed and evaluated for pulmonary metastases (Fig. 4b, e). *m*-Tyr treatment drastically decreased the number of lung metastases as compared to controls. The Kaplan–Meier overall survival curves show the significant difference in the percentage of survivors for *m*-Tyr-treated and control mice as a function of the days after tumor cell injection (Fig. 4c, f).

**Fig. 5:**
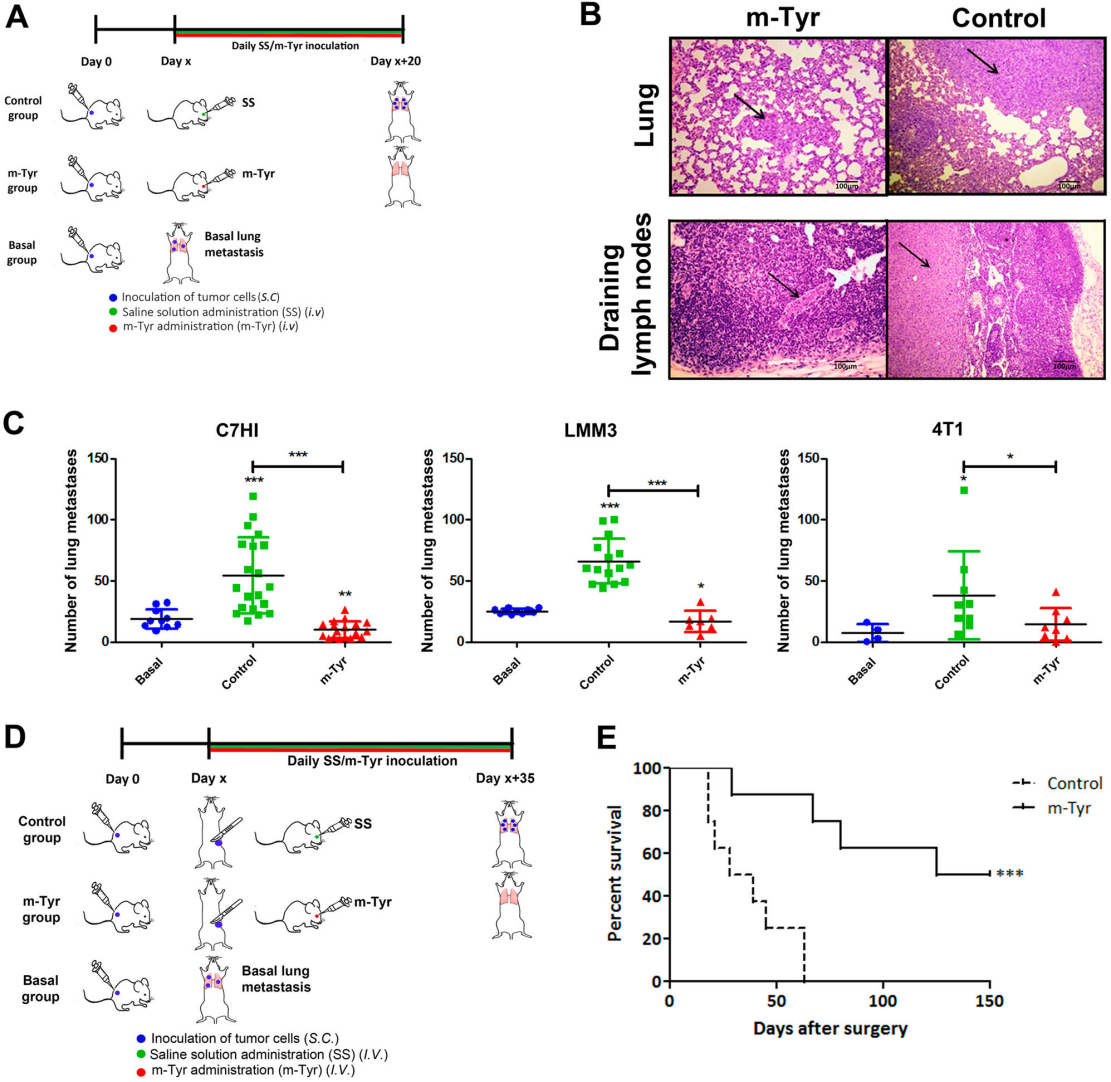
*m*-Tyr inhibits spontaneous metastasis **a** Schematic representation of the in vivo assay carried out to assess the role of m-Tyr in spontaneous metastasis using three metastatic murine mammary carcinomas C7HI, LMM3, and 4T1. For C7HI tumors, BALB/c mice were s.c. injected with 2 × 10^5^ cells and 40 days later animals received a daily i.v. injection of *m*-Tyr (67 mg/kg/day) or saline for the following 20 consecutive days. A third group was killed at day 40 to evaluate the number of metastases at the onset of treatment (basal). At day 60, all treated and control mice were killed and metastases counted. For LMM3 tumors and 4T1 tumors, BALB/c mice were s.c. injected with 2 × 10^5^ cells and 25 days later animals received a daily i.v. injection of *m*-Tyr or saline as previously described. A third group was killed at day 25 to evaluate the number of metastases at the onset of treatment (basal). At day 45, all treated and control mice were EUTHANIZE and metastases counted. All experiments were done in triplicates. **b** Representative H&E staining of pulmonary and draining lymph nodes metastasis of mice bearing s.c. LMM3 tumors (×25, Scale bar = 100 μm). Arrows point sites of tumor cells in the same field. **c** Pulmonary C7HI, LMM3, or 4T1 metastases for *m*-Tyr treatment. **d** Schematic representation of the in vivo assay carried out to assess the role of *m*-Tyr effect on the survival of LMM3-excised mice exhibiting established lung metastases at the time of surgery. BALB/c mice were s.c. inoculated with 2 × 10^5^ LMM3 tumor cells. Twenty-five days later, tumor-bearing mice were surgically operated to remove the tumor and the remaining mice were killed to evaluate the number of lung metastases at the time of surgery. Tumor-excised mice were divided into two groups: *m*-Tyr (daily i.v. injection of *m*-Tyr, 67 mg/kg, for consecutive 35 days) and control group (daily i.v. injection of saline for consecutive 35 days). **e** Kaplan–Meier estimator for survival indicating the percentage of survivors for *m*-Tyr-treated vs. control mice as a function of the days after tumor excision. *P < 0.05, **P < 0.01, ***P < 0.001

We extended our studies to assess the inhibitory effect of *m*-Tyr on spontaneous metastasis. Using metastatic murine mammary carcinomas 4T1, C7HI, and LMM3, we analyzed the dose–response of *m*-Tyr administration. BALB/c mice were s.c. injected with the different carcinoma cells and when tumor volume reached 400 mm^3^ mice received either *m*-Tyr or saline for the following 20 consecutive days. An additional group was killed at the onset of treatment to evaluate the basal number of metastases. The rest of the groups were subsequently euthanized and lung and hepatic metastases were counted (Fig. 5a–c, Supplementary Fig. 3). Figure 5b depicts representative hemotoxylin and eosin (H&E) staining of pulmonary and draining lymph node metastasis of mice bearing s.c. LMM3 tumors. A significant reduction in the number of lung metastases by *m*-Tyr treatment was detected for the three experimental models (Fig. 5c).

Further, we assessed the *m*-Tyr effect on the survival of LMM3-excised mice exhibiting established lung metastases at the time of surgery. BALB/c mice were s.c. inoculated with LMM3 tumor cells. Twenty-five days later, tumor-bearing mice were operated to remove the tumor and the remaining mice, were killed to evaluate the number of lung metastases at the time of surgery (mean (range) = 18 (12–25)). Next, tumor-excised mice received daily i.v. either *m*-Tyr or saline for the following 35 consecutive days (Fig. 5e). No tumor relapse at the site of inoculation was seen after surgical excision. The Kaplan–Meier estimator of the overall survival curves evidenced a significant difference in the percentage of survivors for *m*-Tyr-treated vs. control mice as a function of time after tumor excision (Fig. 5f). While all control mice died exhibiting a high number of lung metastases at 36.9 ± 6.7 days (mean ± S.E.M.) after surgery, in contrast, 50% of mice died in the *m*-Tyr group, while the other 50% remained alive without exhibiting signs of local or metastatic disease in the long run (Fig. 5f). Further, when these mice were killed 18 months after the end of the treatment, no metastatic foci were detected.

The analysis of potential toxic side effects associated with *m*-Tyr periodic administration (45 days) revealed neither histologic nor cytologic alterations, even when organs with high rate of renewal (skin, bone marrow, or small intestine) were studied (Supplementary Fig. 4). Leukocyte subsets in both draining lymph nodes and the spleen were not altered (Supplementary Fig. 4). Accordingly, *m*-Tyr-treated mice did not display significant changes in either the humoral or the cellular immune responses, observed by the titer of sheep red blood cell-specific antibodies and delayed hypersensitivity against ovoalbumin, respectively, and did not exhibit any other detectable functional alterations assessed by the number of erythrocytes, leukocytes, and platelets in the blood and the concentration of circulating hepatic GOT and GPT transaminases, total proteins, urea, creatinine, alkaline phosphatase, cholesterol, and glucose.

### In vitro effect of *m*-Tyr

**Fig. 6:**
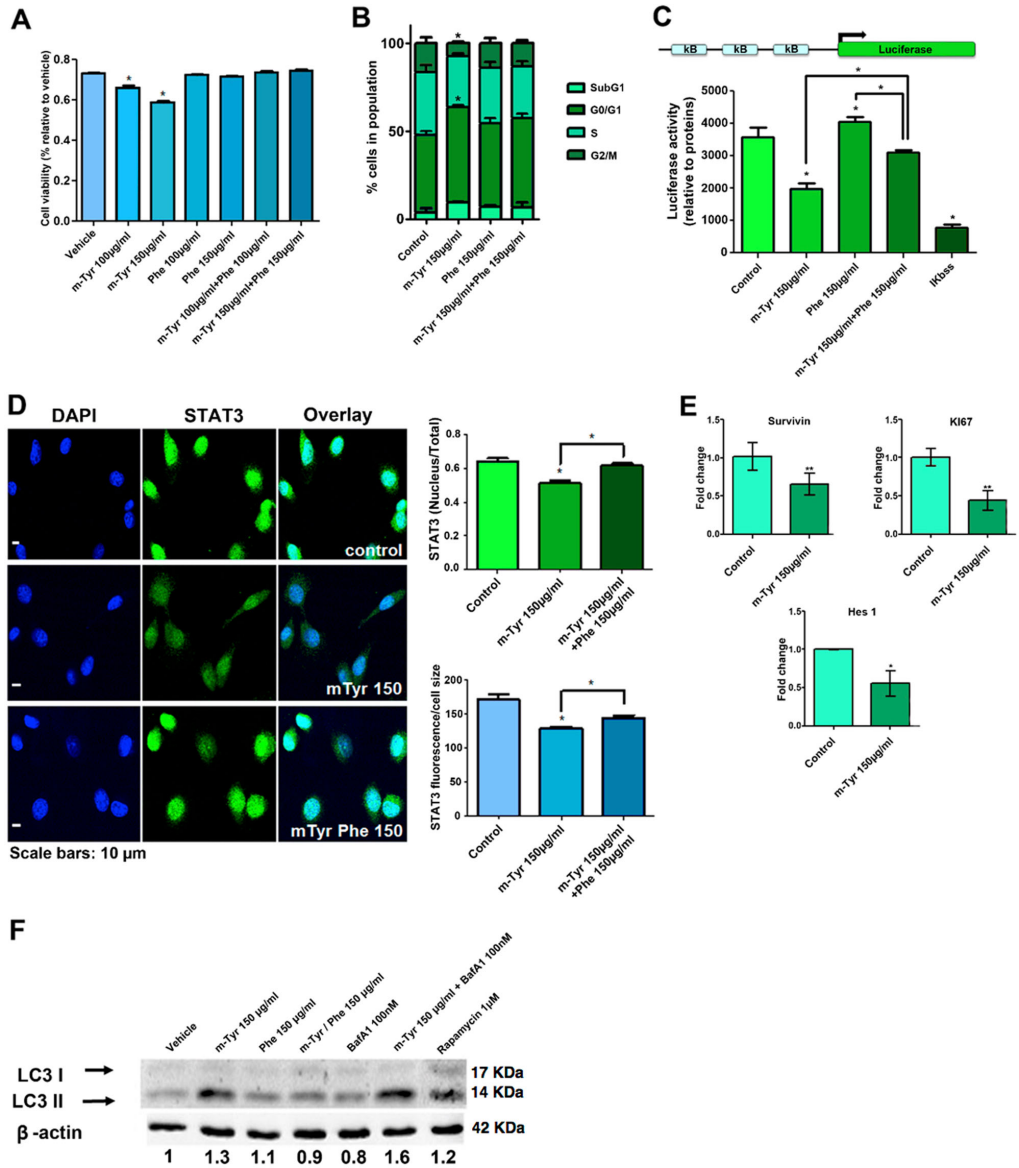
In vitro* m*-Tyr downregulates key signaling pathways in PCa **a** PC3 cell survival was assessed by the colorimetric MTS assay. Cells were cultured in the presence of *m*-Tyr (100l or 150 μg/ml; 24 h) and/or Phe (100 or 150 μg/ml; 24 h). Results are expressed as the viability percentage relative to the control in the presence of the vehicle (100%) ± S.E.M. (*P < 0.05, significant difference). **b** Effect of *m*-Tyr (150 μg/ml; 24 h) and/or Phe (150 μg/ml; 24 h) on cell cycle progression in PC3 cells. Once treated, cells were processed for DNA staining with propidium iodide. Flow cytometry analysis was performed for cell cycle distribution. The percentage of cells in different phases of cell cycle was determined by ModFit LT cell cycle analysis software (left panel). The right panel shows the percentage relative to control of cells in each population. Data shows the mean of five independent experiments ± S.D.. **c** PC3 cells were transfected with a NFκB—responsive luciferase reporter construct and were exposed to *m*-Tyr (150 μg/ml, 24 h) and/or Phe (150 μg/ml, 24 h). Ikbss was used as a negative control. **d** PC3 cells were cultured in the presence of *m*-Tyr (150 μg/ml; 24 h), *m*-Tyr + Phe (150 μg/ml; 24 h) or vehicle. Cells were fixed and stained with anti-STAT3 primary antibody and a secondary antibody conjugated to Alexa Fluor 488. Segmentation of the whole cell and nucleus (DAPI) was performed to calculate the ratio of STAT3n/STAT3t in a cell-by-cell analysis and the florescence intensity for STAT3 was calculated using Matlab and normalized to the cell size (*n* ≥ 20 cells for each condition). (*P < 0.05, significant difference). **e** PC3 cells were pre-treated with different concentrations of *m*-Tyr (150 μg/ml; 24 h) or control (Ctrol). Total RNA was extracted and the expression levels of mRNA for each gene, were determined by RT-qPCR. Data were normalized to cyclophilin-A. One representative from at least three independent experiments is shown. **f** Autophagy levels in response to *m*-Tyr. PC3 cells were treated with *m*-Tyr (150 μg/ml), Phe (150 μg/ml) or (*m*-Tyr/ Phe 150 μg/ml) for 24 h. Cells were treated with Bafilomycin A1 (BafA1 100 nM) 2 h before collecting. Treatment with rapamycin was used as a positive control (1 μ for 6 h). Western blot was performed with specific antibodies against for LC3I and LC3II (left panel). Densitometry analysis was performed (right panel). *P < 0.05, **P < 0.01 significant difference

To explore *m*-Tyr cellular effects in vitro, we assessed *m*-Tyr and/or Phe (100 and 150 μg/ml) effect on cell growth and cell cycle progression in the PCa cell line, PC3. *m*-Tyr significantly reduced cell viability in a dose-dependent manner, effect that was completely reversed by the addition of Phe in equivalent concentrations. Phe alone had no effect on PC3 cell viability (Fig. 6a). Additionally, *m*-Tyr (150 μg/ml) produced a significant arrest of PC3 cells in G_0_/G_1_ phase (Fig. 6b). This increase in G_0_/G_1_ cell population was mostly at the expense of the S phase. Accordingly, this effect was counteracted by the addition of Phe in equivalent concentrations. Phe alone had no effect on the distribution of the cell cycle population (Fig. 6b).

To assess *m*-Tyr cellular effects at the molecular level, we evaluated NFκB activity. NFκB is constitutively activated in human PCa and correlates with disease progression^14^. Using a reporter construct containing five repeats of the NF-KB consensus binding sequence cloned upstream of the luciferase gene (pNFκB-luc), we showed that *m*-Tyr significantly repressed (45.14%, *P < 0.05) the transcriptional activity of the NFκB-luc in PC3 cells (Fig. 6c). Phe addition partially reversed the inhibition produced by *m*-Tyr but no alterations were observed in the reporter activity by Phe alone (Fig. 6c). For control purposes, we transfected PC3 cells with the mutated inhibitor IkBss confirming the NFκB transactivation inhibition (78.24%, *P < 0.05, Fig. 6c).

Given the tight association between STAT3 and PCa and that NFκB and STAT3 are two oncogenic transcriptional factors activated simultaneously and cooperatively inducing various survival factors^15^, we next evaluated the effect of *m*-Tyr and Phe on STAT3 activation. As the role of STAT3 as a DNA-binding transcription factor depends on its ability to gain entrance to the nucleus, STAT3 cellular localization in PC3 cells treated with *m*-Tyr were imaged by confocal microscopy. The images displayed in Fig. 5d showed that *m*-Tyr reduced STAT3 expression and nuclear localization. Phe addition partially restored both parameters (Fig. 6d). By reverse transcription quantitative polymerase chain reaction (RT-qPCR), we assessed survivin expression (downstream target of STAT3) and confirmed its downregulation by *m*-Tyr (Fig. 6e). Further we also detected significantly lowered *MKI67* mRNA levels in *m*-Tyr-treated PC3 cells compared to controls (Fig. 6e).

The constitutive activation of STAT3/NFκB signaling can regulate the Notch pathway, which appears to play a key role in a variety of cancers and controls cell fate determination, survival, proliferation, and the maintenance of stem cells^16–18^. Hence, we also screened for molecular targets associated with the Notch pathway. Results showed a significant downregulation of *HES1* mRNA levels (44.6%, *P < 0.05) when cells were exposed to *m*-Tyr (Fig. 6e).

Previous reports show that STAT3 inhibition induces signs of autophagy^19^. Moreover, *m-*Tyr (ROS-damaged phenylalanine) may be incorporated into eukaryotic proteins via a specific tRNA-dependent pathway, using mitochondrial and possibly cytosolic phenylalanyl-tRNA synthetase^20^ and elevated *m*-Tyr content in proteins may lead to the dysfunction of intracellular signaling and activation of autophagy. To identify *m*-Tyr as a novel inducer of autophagy in PCa, we assessed LC3 lipidation indicated by the conversion of LC3-I into LC3-II. Results show that endogenous levels of LC3-II accumulated upon *m*-Tyr treatment (Fig. 6f). This effect was counteracted by the addition of Phe. No detectable levels of LC3-II were observed for Phe treatment alone (Fig. 6f). We also examined if autophagosomes were fusing with lysosomes into autophagolysosomes under *m*-Tyr, adding bafilomycin A1 (BafA1)^21^. Results showed LC3-II accumulation upon exposure of cells to *m*-Tyr in the presence of BafA1, strongly suggesting that *m*-Tyr induced autophagosome formation and that the autophagic pathway was functional (Fig. 6f).

## Discussion

Surgical removal is one of the main treatments for solid tumors. However, although it is recommended in most clinical cases, tumor removal may trigger the acceleration of regional and distant metastases. This suggests that the primary tumor could inhibit the growth of its own metastases, an event that is mimicked by the phenomenon of CR induced in experimental animal models^9^.

Michelson and Leith^22^ attempted to provide mathematical modeling explanation rather than a biological one to address the occurrence of CR. However, these authors failed to elucidate the central paradox of CR, that is, the inhibition of secondary tumor implants together with the progressive growth of the primary tumor, because in their mathematical constructs, they did not offer a plausible explanation for the fact that inhibitory factors produced by the primary tumor could not affect it.

Here, we propose a novel therapeutic option for metastatic tumors. We discovered that *m-*Tyr treatment significantly abolished the proliferative capacity of cancer cells and further impaired tumor growth when cells were xenotransplanted in *nu/nu* in experimental human cancer models. Accordingly, Phe, a protective amino acid highly present in primary tumors and precursor of *m-*Tyr, was capable of reversing the effects caused by *m-*Ty*r*.

Gurer-orhan et al.^23^ reported *m-*Tyr incorporation into proteins by CHO (Chinese-hamster ovary) cells and that it impaired CHO cell viability. They suggested that this *m-*Tyr might be misincorporated into proteins during translation and that this mechanism could contribute to its toxicity. Further *m*-Tyr has been reported as a potent, structurally unusual broad-spectrum phytotoxin exuded by the roots of some fine leaf fescue grasses that allow them to outcompete or displace neighboring plants^24^.

The specific pathway by which ROS-damaged amino acids are incorporated into proteins remains unclear. However, Klipcan et al.^25^ provided evidence that phenylalanyl-tRNA synthetases (PheRS) catalyze the direct attachment of *m-*Tyr to tRNAPhe, delivering the misacylated tRNA to the ribosome and enabling the incorporation of the ROS-damaged amino acid into eukaryotic proteins. Crystal complexes of PheRSs with *m-*Tyr describe the net of highly specific interactions within the synthetic and editing sites.

Accumulation of damaged proteins evidences the inefficient proteolytic machinery. This is commonly seen by the increased amounts of aggregated, misfolded, and oxidized proteins that accumulate in atherosclerosis, neurodegeneration, and cancer. Mildly oxidized proteins are usually degraded by the proteasome system^26^. However, in the case of abundant oxidized proteins, there are evidences pointing to the autophagy of such proteins^27^.

Our results showcase *m-*Tyr as a novel inducer of autophagy in PCa. PC3 cells exposed to *m-*Tyr display accumulated levels of LC3-II, effect counteracted by the addition of Phe. Further, the addition of BafA1 confirmed the autophagosome formation by *m-*Tyr and the functionality of the autophagic pathway. The autophagic scenario in carcinogenesis, metastasis, and cancer therapy is highly complex with reports demonstrating functions in tumor promotion as well as in tumor suppression and a potential contribution to therapeutic resistance^28^. Moreover, the high level of heterogeneity within a tumor adds a greater degree of complexity to whether this cellular mechanism may favor or not tumor dormancy^29^.

Next, we explored *m-*Tyr impact on the STAT3/NFκB/Notch signaling cascade. This axis is activated in several tumor types^30^, and is also associated with the autophagy process and of particular relevance in PCa^16–18^. Our data showed that *m-*Tyr impaired the STAT3/NFκB/Notch pathway. STAT3 has recently been considered a new autophagy regulator^31^. Thus, *m-*Tyr impact on autophagy may be in part due to STAT3 downmodulation and in turn blocking this pathway may impair tumor growth and progression.

Strikingly, our results show that *m-*Tyr almost completely abolished the number of experimental metastases of PCa and even spontaneous metastasis of breast cancer experimental models. Further, in the case of *m-*Tyr effect on the survival of LMM3-tumor-excised mice exhibiting established lung metastases at the time of surgery, we observed no tumor relapse after surgical excision and a significant increase in the percentage of survivors for *m-*Tyr-treated animals vs. control mice. These results demonstrate that *m-*Tyr not only inhibits the implantation of new metastases of mice and human derived xenografts, but also is able to impair growth of cells from the primary tumor that may have remained post surgery. Potential toxic side effect analysis associated with a *m-*Tyr periodic administration revealed neither histologic nor cytologic alterations, even when organs with high rate of renewal were studied. Leukocyte subpopulations in both the draining lymph nodes and the spleen remain unchanged and neither the humoral nor the cellular immune responses displayed significant changes nor any alteration in different functional patterns in *m-*Tyr-treated mice. Recently^2^, we have suggested that the apparent lack of inhibitory effect of *m-*Tyr on normal proliferating tissues might be explained by assuming that these tissues display—as primary tumors do—a content of Phe high enough to counteract the inhibitory effects produced by *m-*Tyr.

Up to date, there is no evidence pointing to *m-*Tyr cytotoxicity in humans. Fell et al.^32^ reported on the metabolism of *m*-Tyr in man. Oral doses of *m-*Tyr (5 mg/kg) were given to adults and the amino acid blood levels measured at intervals following the load. The majority of the load was metabolized to m-hydroxyphenylacetic acid, m-hydroxymandelic acid, and 3,4-dihydroxyphenylacetic acid detected in urine samples. No detectable toxic side effects were observed.

Our data suggest that, for therapeutic purposes, *m-*Tyr has many attractive features, exerting its anti-metastatic effect at low concentrations with no detectable toxic side effects. Delivery of this biologically active metabolite may boost the therapeutic efficacy of the current treatment of cancer metastasis, specifically by controlling the outburst of micrometastases following removal of a primary tumor, or other stressors that might trigger the escape of metastases from dormancy.

## Materials and methods

### Cell culture, treatments, reagents, and antibodies

PC3, Calu6, and KB cells were obtained from the American Type Culture Collection (Manassas, VA, USA) and were routinely cultured in RPMI 1640 (Invitrogen, Grand Island, NY, USA) supplemented with 10% fetal bovine serum (FBS).

The mammary carcinoma cell lines C7HI and LMM3, were kindly provided by Dr. C. Lanari (Instituto de Biología y Medicina Experimental, Buenos Aires, Argentina, IBYME) and Dr. L. Colombo (Instituto Ángel Roffo, Buenos Aires, Argentina), respectively. The 4T1 cell line was provided by Dr. N. Zwirner (IBYME). *m*-Tyrosine (*m*-Tyr) and phenylalanine (Phe) was obtained from Sigma-Aldrich (San Luis, MO, USA). For treatments, cells were incubated 24 h in RPMI media containing 10% FBS and then were exposed to meta-tyrosine (*m*-Tyr) or phenylalanine (Phe) (100 and 150 μg/ml, 24 h).

Polyclonal anti-STAT3 antibody was purchased from Stressgen Biotechnologies Corp. (San Diego, CA). Anti-β-Actin antibody was purchased from Sigma-Aldrich (Gillingham, Dorset, UK). Anti-mouse and anti-rabbit secondary antibodies conjugated with horseradish peroxidase (HRP) were from Amersham Ltd. (Freiberg, Germany). Secondary antibodies conjugated with Alexa Fluor 488 or Alexa Fluor 555 were from Molecular Probes and Invitrogen.

### Animals

Eight to 10-week-old male athymic (*nu/nu*) each weighing at least 20 g, were purchased from CONEA (Comisión Nacional de Energía Atómica/Centro Atómico Ezeiza. Aplicaciones Tecnológicas y Agropecuarias/Bioterio, Buenos Aires, Argentina). BALB/c mice, 3–5-month-old were obtained from the animal core facility at IMEX-CONICET. Mice were used in accordance with the “Guidelines for the Welfare of Animals in Experimental Neoplasia” (UK Coordinating Committee on Cancer Research) and the NIH Guide and Use of Laboratory Animals. Protocol number 025/23016 approved by the Institutional Committee for the Care of Laboratory Animals (CICUAL), Institute of Experimental Medicine, CONICET.

### Concomitant resistance experiments

Eight to 10-week-old male athymic *(nu/nu*) mice were randomized into two groups. Human PCa cells were injected s.c. in the right flank of the experimental group (primary tumor-bearing mice) and, at selected times (7, 14, or 21 days) after tumor inoculation—when PC tumor volumes were 101 ± 17, 317 ± 42, or 317 ± 42 mm^3^ (mean ± S.E.), respectively—a secondary tumor implant was carried out in the left flank. Control mice only received the tumor implant in the left flank. Body weight and tumor growth were measured every 2 days starting at 8 days after inoculation when tumors became palpable under the skin. Their volumes were calculated using the formula 0.4 (*a* × *b*^2^), where *a* and *b* represent the larger and smaller tumor diameters, respectively^10^.

In experiments where *m*-Tyr and Phe, animals were randomly assigned into four groups: control; experimental (CR); Phe and *m*-Tyr. Tumor cells (5 × 10^6^) were s.c. inoculated (first challenge) in the right flank of animals of the CR and Phe groups. A total of 3 × 10^6^ cells were inoculated in the left flank of the animals of all groups 14 days after the first challenge. Twenty-four hours later, both, Phe and *m*-Tyr groups were injected in the same site with Phe or *m*-Tyr (0.2 ml of 500 mg/ml) respectively, for 15 days on a daily basis. Tumor growth was measured every 2 days starting 8 days after inoculation when the tumors were palpable. Their volumes were calculated as described above.

The generation of Calu-6 and KB experimental tumors is described in Supplemental materials and methods.

### Experimental and spontaneous metastasis

#### Experimental metastases of human tumor cells

For experimental PCa metastasis, nude mice (*n* = 9) were i.v. challenged with 2 × 10^6^ PC3 tumor cells, and were subsequently injected with *m*-Tyr or saline on a daily basis (i.v. administration; 67 mg/kg/day). Animals were killed 20–25 days after tumor cell inoculation and evaluated for pulmonary metastases or left to assess survival. All experiments were done in triplicates.

#### Spontaneous metastases in tumor-bearing mice

For the analysis of the growth of spontaneous metastasis, the highly metastatic murine mammary carcinomas 4T1, C7HI, and LMM3 were used. In the case of C7HI, BALB/c mice were s.c. injected with 2 × 10^5^ cells. Forty days later, mice were divided in three groups that received, between days 40 and 60, a daily i.v. injection of of *m*-Tyr (67 mg/kg, *n* = 18) or saline solution (control, *n* = 20). A third group (*n* = 10) was killed at day 40 to evaluate the number of metastases at the onset of treatment. At day 60, all mice were killed and lung and hepatic metastases were counted. All experiments were done in triplicates.

For LMM3 tumors, BALB/c mice were s.c. injected with 2 × 10^5^ cells. Twenty-five days later, animals were divided in three groups that received, between 25 and 45 days, a daily i.v. injection of *m*-Tyr (67 mg/kg, *n* = 7, respectively) or saline (*n* = 15). A third group (*n* = 10) was killed at day 25 to evaluate the number of metastases at the onset of treatment. At day 45, all treated and control mice were euthanized and metastases. All experiments were done in triplicates.

For 4T1 tumors, BALB/c mice were s.c. injected with 2 × 10^5^ cells. Twenty-five days later animals were divided into two groups that received, between 25 and 45 days, a daily i.v. injection of *m*-Tyr (67 mg/kg; *n* = 8) or saline (*n* = 9). A third group (*n* = 4) was killed at day 25 to evaluate the number of metastases at the onset of treatment. At day 45, all treated and control mice were killed and metastases counted. All experiments were done in triplicates.

#### Spontaneous metastases after surgical extirpation of the primary tumor

BALB/c mice (*n* = 22) were inoculated s.c. with 2 × 10^5^ LMM3 cells. Twenty-five days later, tumors were surgically removed from 18 tumor-bearing mice; the remaining six animals were killed to evaluate the number of lung metastases at the time of surgery. Lung metastases were counted as described in ref. ^33^. Then, the tumor-excised mice were divided into two groups. One group (*n* = 8) received, for the following consecutive 35 days, a daily i.v. injection of *m*-Tyr (67 mg/kg). The other group (*n* = 8) received saline. All experiments were done in triplicates.

### Histopathologic studies

The highest dose of *m*-Tyr (67 mg/kg) used in experiments was daily administered by the i.v. route for 45 days in BALB/c mice. At day 45, a sample of mice was euthanized and the following organs—skin, liver, kidney, spleen, lung, bone marrow, small and large intestine—were investigated histopathologically. Samples were fixed as previously reported^7^.

### Luciferase assay

PC3 cells were seeded on 12-well plates (1.2 × 10^5^ cells per well) and transfected with a NFκB—responsive luciferase reporter construct (2 µg) and/or Ikbss expression vector (2 µg) as a negative control using polietilenimine (Sigma-Aldrich, UK). Cells were treated with *m*-Tyr (150 µg/ml, 24 h) and/or Phe (150 µg/ml, 24 h). The luciferase assay system (Promega, Madison, WI) was used to measure luciferase activity in a Glomax luminometer (Promega). Transfections were performed in triplicate and each experiment was repeated at least three times. Data were normalized to total protein determined by Bradford assay.

### Immunofluorescence experiments and quantitative microscopy

Cells were seeded in 12-well plate at a density of 1 × 10^5^ cells per well on coverslips overnight. Cells were treated with *m*-Tyr (150 μg/ml; 24 h), *m*-Tyr + Phe (150 μg/ml; 24 h) or vehicle, fixed in ice-cold methanol, permeabilized for 10 min with 0.5% Triton X-100/phosphate-buffered saline (PBS), washed twice with PBS and then blocked with 5% bovine serum albumin (BSA) in PBS. Cells were incubated overnight with an anti-STAT3 antibody diluted 1:200 in 3% PBS and 0.1% BSA. Cells were washed with PBS and incubated with fluorescent secondary antibodies Alexa 647 (1:3000) for 2 h. Negative controls were carried out using PBS instead of primary antibodies. Cells were washed, mounted in FluorSave Reagent (Merck Millipore), and imaged by confocal laser scanning microscopy with an Olympus Fluoview FV 1000 microscope (and its software). An Olympus water immersion ×60 objective (1.20 N.A. UPLAN APO) objective was used. Samples were imaged at room temperature. Wide field microscopy was carried on using an Olympus IX71 microscope with an Olympus UApo water immersion ×40 objective (1.15 N.A.), a mercury arc lamp excitation, and suitable filters. Camera: Hamamatsu Orca CCD C4742-95. Samples were imaged at room temperature.

### Image processing for presentation

Confocal and wide field microscope images were processed for presentation using ImageJ software. Background of each channel was subtracted and in some cases a median filter (radius: 1 pixel) was applied only for presentation

### RNA isolation and RT-qPCR

Total RNA was isolated with the RNeasy Mini Kit (Qiagen). Complementary DNAs (cDNAs) were synthesized with RevertAidTM Premium First Strand cDNA Synthesis Kit (Fermentas) and used for real-time PCR amplification with Taq DNA Polymerase (Invitrogen) in a DNA Engine Opticon (MJ Research). Each PCR was performed in triplicate and three biological independent experiments were performed. Primers were designed to amplify a 100 bp region present in the fully mature RNA species of Survivin (Fw: 5′-GGAGCCAGATGACGACCCCA-3′ and Rv: 5′-AGCGCAACCGGACGAATGCT-3′), Hes (Fw: 5′-GTGAAGCACCTCCGGAAC-3′ and Rv: 5′-CGTTCATGCACTCGCTGA-3′), Ki67 (Fw: 5′-CCAGCACGTCGTGTCTCAAGAT-3′ and Rv: 5′-ACACTGTCTTTTGAGTCATCTGCGG-3′), STAT3 (Fw: 5′-TCGCAGCCGAGGGAACAAG-3′ and Rv: 5′-GCCATCCTGCTAAAATCAGGGG-3′), ACTB (Fw: 5′-CGGTTGGCCTTAGGGTTCAGGGGGG-3′ and Rv 5′-GTGGGCCGCTCTAGGCACCA-3′), and GAPDH (Fw: 5′-CAGTCAGCCGCATCTTCTTTTG-3′ and Rv: 5′-ACCAGAGTTAAAAGCAGCCCT-3′). Data were analyzed by Opticon-3 software and normalized to β-actin or GAPDH. Errors were calculated as in ref. ^34^.

### Cell viability

Cell viability was assayed by MTS (Cell Titer 96 wells aqueous non-radioactive Cell proliferation assay, Promega) following the manufacturer’s instructions. Each sample was done in triplicate in five independent experiments.

### Cell cycle analysis

PC3 stable cell lines were treated with *m*-Tyr or Phe (150 μg/ml; 24 h), *m*-Tyr + Phe (150 μg/ml; 24 h) or vehicle, and then stained with propidium iodide (PI) and analyzed by fluorescence-activated cell sorting (FACS). After 24 h exposure of the PC3 cell line to the compounds, cells were collected by trypsinization and gently pelleted by centrifugation at 3000 r.p.m. for 3 min. Cells were resuspended in cold PBS and centrifuged again (3000 r.p.m. for 3 min). Pellets were transferred dropwise to 1 ml of 70% (v/v) ethanol, allowed to fix for 2 h at 4 °C, and kept on ice. The ethanol-suspended cells were collected, washed, and resuspended in 1 ml PBS containing DNase-free RNase A (0.2 mg/ml), Triton X-100 (0.1% v/v), and PI (0.02 mg/ml). Each sample was then incubated at 37 °C for 15 min before cell cycle analysis with a BD flow cytometer and FlowJo 7.6.2 software.

### [3H]-thymidine uptake assay

A total of 3 × 10^5^ tumor cells in 0.1 ml of medium were cultured with 0.1 ml of several twofold dilutions of serum and 1 mCi per ml of [3H]-thymidine (Dupont NEN Research Products) as described^6^. After 18 h, radioactivity incorporated into the cells was determined in a β counter (Beckman). The titer of growth-inhibitory activity was defined as the reciprocal of the serum dilution, producing 50% inhibition of [3H]-thymidine uptake by tumor cells as compared with [3H]-thymidine uptake of tumor cells incubated with medium only and expressed as growth-inhibitory units 50 by ml (GIU50 per ml). Results are expressed as mean ± S.E.M. of three samples of normal serum, three samples of serum from mice bearing large PC3 tumors (1700 mm^3^ approximately), and three samples of serum from mice bearing small PC3 tumors (500 mm^3^ approximately). Each dilution of each serum was assayed in triplicate. *P < 0.05; ***P < 0.001 significant difference.

### Serum and tyrosine purification

Serum was prepared as described in refs. ^6,35^. Serum was then subjected to dialysis using a dialysis membrane with a 1 KD cutoff (width 2.5 cm) for 48 h. The dialyzable fractions were lyophilized and resuspended in a mixture of water:methanol:trifluoroacetic (87:12:1) for tyrosine determination. *p*-tyrosine (*p*-tyr), *m-Tyr*osine (*m*-tyr), and *o*-tyrosine (*o*-tyr) determinations in the dialyzable fractions from normal serum as well as serum from mice bearing PC3 or Calu6 tumors were carried out using an HPLC. Methodology is previously described in ref. ^10^. Briefly p-Tyr, *m*-Tyr, and *o*-Tyr separation was performed in a C-18 Zorbax column (Agilent Santa Clara, CA, 7.5 mm × 4.6 mm i.d., 5 μm particle size). The mobile phase consisted in a mixture of water:methanol:trifluoroacetic (87:12:1), the flow rate was set at 0.5 ml/min, and the UV detection was performed at 280 nm. The retention times for p-Tyr, *m*-Tyr, and o-Tyr were: 2.12 min; 2.55 min; 3.34 min, respectively. Standards for p-Tyr, *m*-Tyr, and o-Tyr purchased from Sigma, Argentina, were used.

### Western blot

Processing of LC3 protein into LC3-II, the phagophore- and autophagosome-associated form was assayed. After 24 h of *m*-Tyr and or *m*-Tyr/Phe stimulation, cells were washed with PBS and protein extracts from fractions were obtained. Protein concentration was determined using the bicinchoninic acid protein assay kit from (Sigma-Aldrich, UK). Western blotting was performed by standard methods using 10% polyacrylamide gels. Each nitrocellulose membrane was blotted with antibodies to LC3 (Cell Signalling Technology, 2775) and B-actin (Sigma). Bound antibodies were detected with horseradish peroxidase-conjugated antibody (Bio-Rad, anti-rabbit 170-6515, anti-mouse 170-6516) using Amersham ECL PLUS reagent (GE Healthcare, RPN2106). Images were obtained with an Intelligent Dark Box (Fujifilm LAS1000, Tokyo, Japan) and analyzed with ImageJ Analysis software. The intensity of each band was expressed as arbitrary units (A.U.).

### Statistical analysis

Results are shown as mean ± S.E.M. unless otherwise is stated. The Student’s *t* test, Mann–Whitney *U*-test, and Kaplan–Meier estimator for survival curves were used. Differences were considered significant when *P* < 0.05.

## Electronic supplementary material

Supplemental figures

supplemental methods for supplemental figures
