# Supplementary material for: Game-changing restraint of Ros-damaged phenylalanine, upon tumor metastasis
Source: Cell Death Dis. 2018 Feb 2;9(2):140. doi: 10.1038/s41419-017-0147-8 (PMC5833805; doi:10.1038/s41419-017-0147-8)
Supplement: Supplementary file 1 — Supplemental figures [file 41419_2017_147_MOESM1_ESM.pdf]

SUPPLEMENTAL FIGURE 1

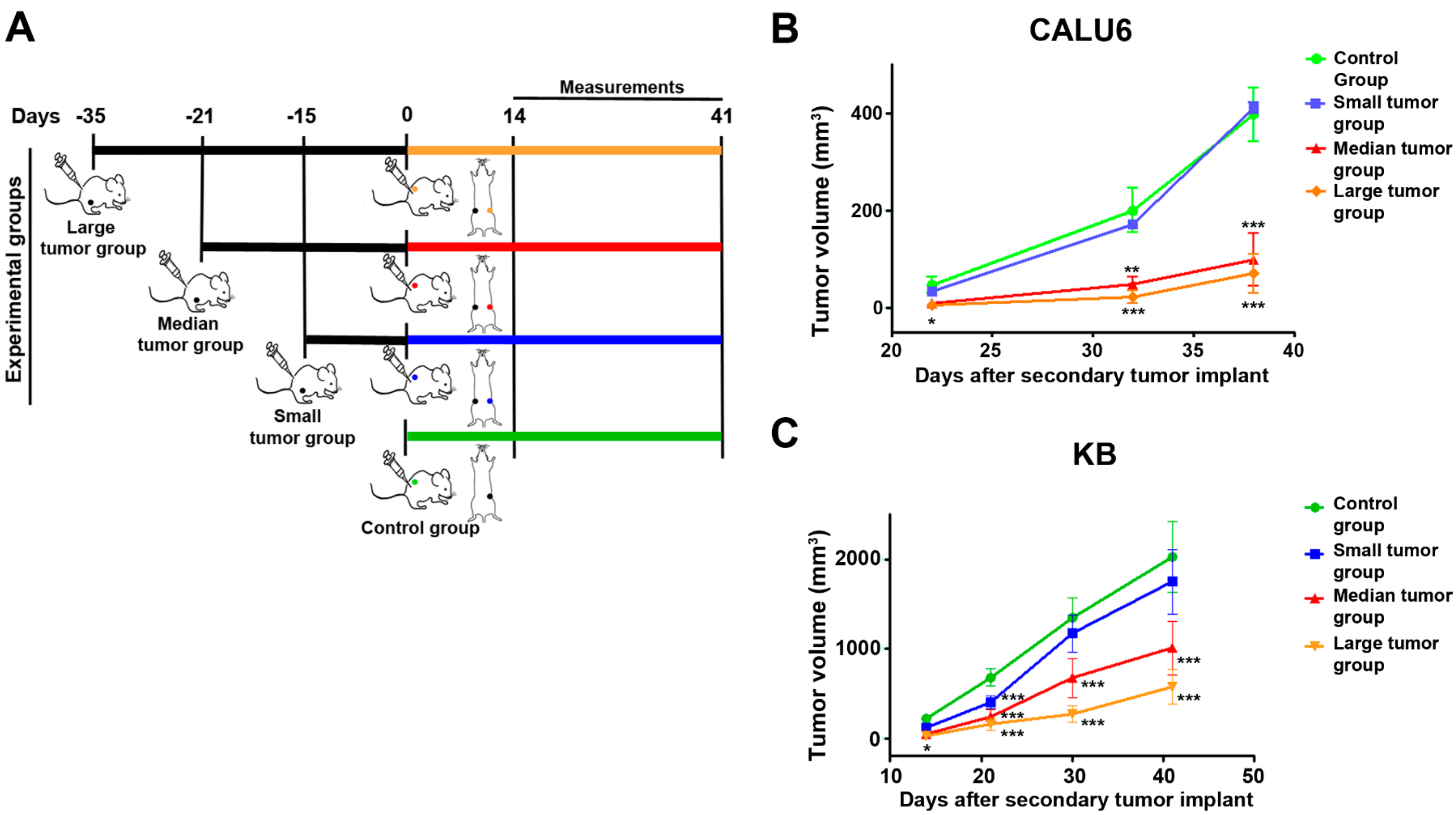

Supplemental Figure 1. Concomitant resistance occurs in Calu6 (lung anaplastic carcinoma) and KB (nasopharyngeal carcinoma) experimental models) A) Schematic representation of CR strategy.8- to 10-wk- old male athymic *(nu/nu)* mice were randomized into four groups: control (n=5) and 3 experimental groups (small, medium and large primary tumors (n=5)). Tumor cells (5 x 10^6) were *s.c.* injected in the right flank at -35, -21 and -15 days of the experimental groups (primary tumors). At day 0 a second inoculation (2 x 10^6 tumor cells) was performed in the left flank of the control and experimental groups (secondary tumors). B & C) Tumor growth measurement began when the tumors became detectable under the skin. The graph shows the average tumor volumes ± S.D. One representative from at least three independent experiments is shown.

SUPPLEMENTAL FIGURE 2

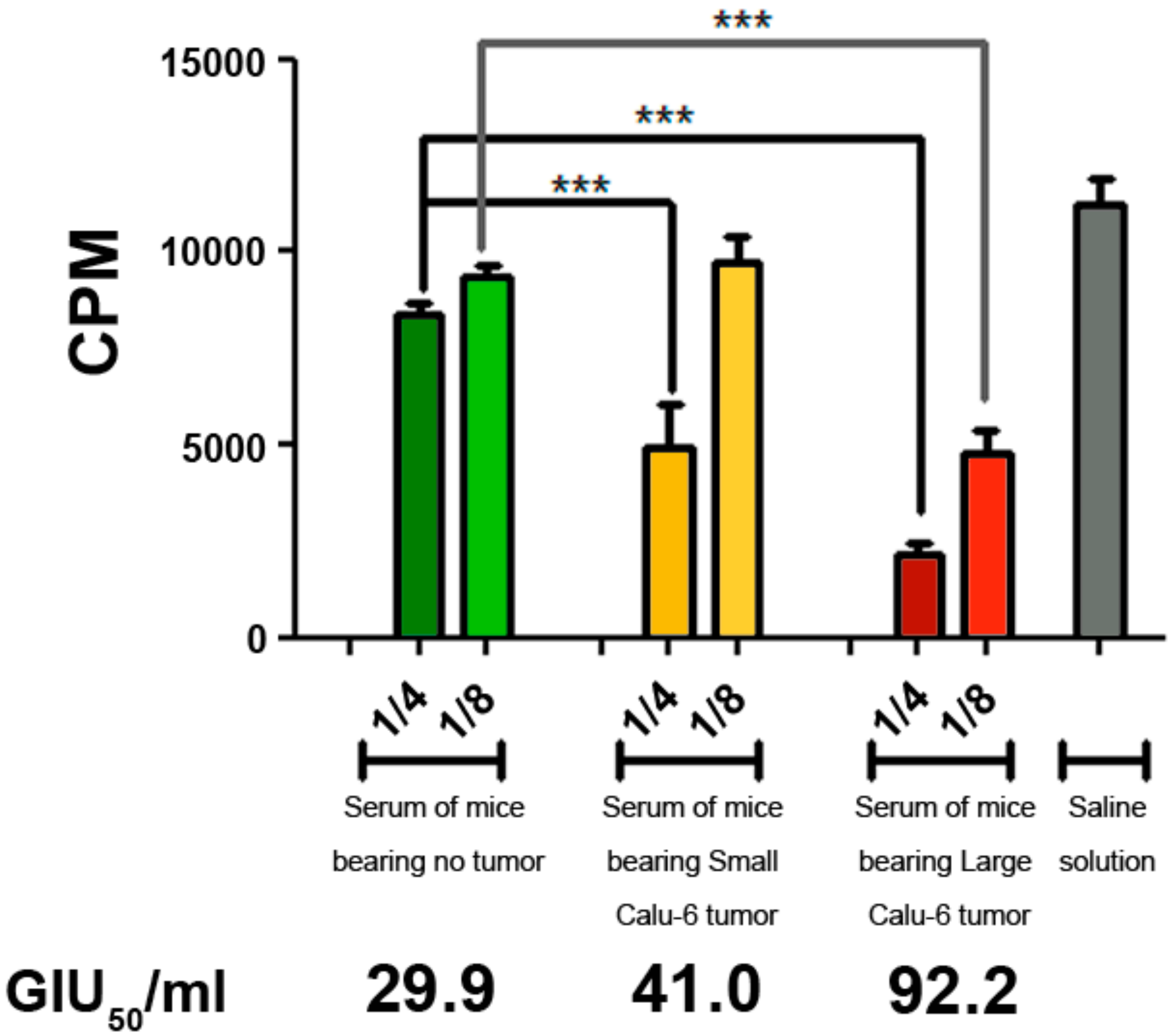

Supplemental Figure 2. Anti-proliferative activity of serum from mice bearing subcutaneous CALU-6 tumors. CALU-6 cells were exposed to different serum dilutions from mice bearing subcutaneous CALU-6 tumors (18 h), and 1 mCi/ml of [3H]-thymidine. Radioactivity incorporated into the cells was determined in a β-counter (Beckman). The titer of growth inhibitory activity was defined as the reciprocal of the serum dilution, producing 50% inhibition of [3H]-thymidine uptake by tumor cells as compared with [3H]-thymidine uptake of tumor cells incubated with medium only and expressed as growth inhibitory units 50 by ml (GIU50/mL). Serum from mice bearing small (100 mm^3) and large (1700 mm^3) Calu-6 tumors presented an elevated GIU_{50}/mL compared with controls. ***P< 0.001 Significant difference.

SUPPLEMENTAL FIGURE 3

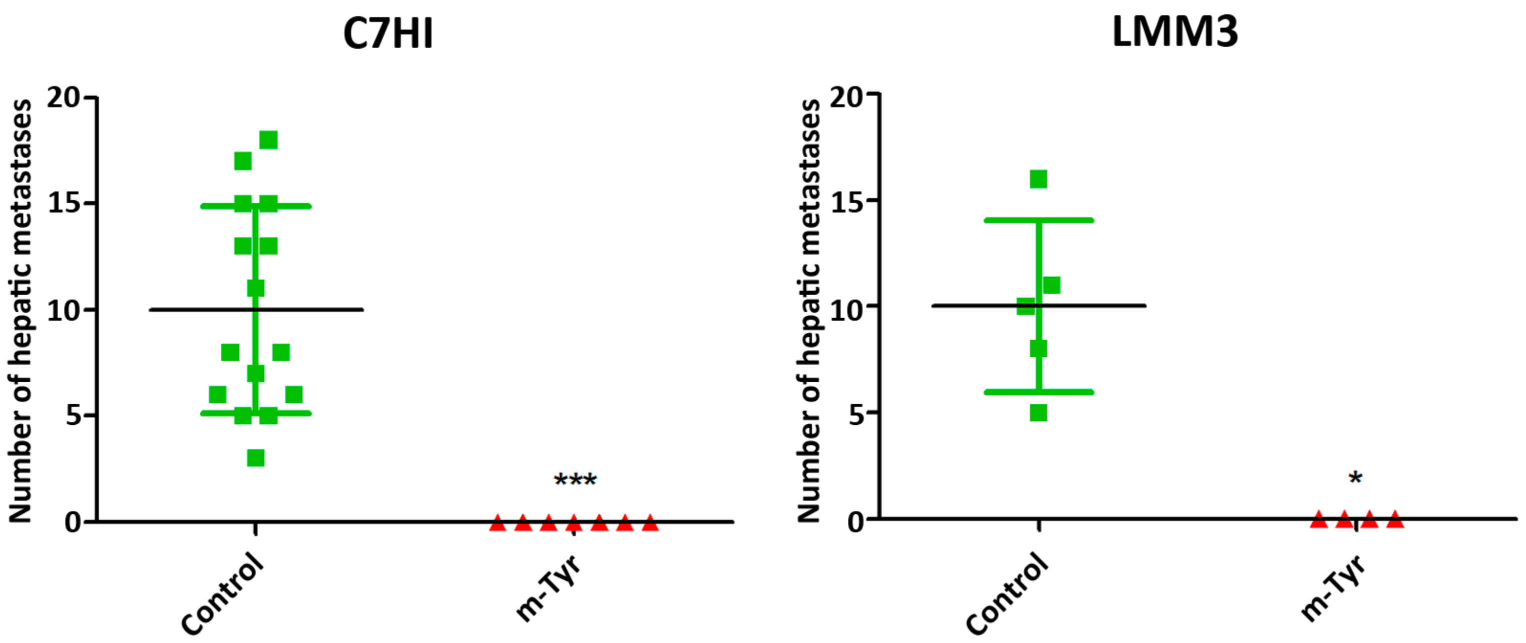

Supplemental Fig 3. **Inhibition of hepatic metastases by m-Tyr in C7HI and LMM3 tumor-bearing mice.** BALB/c mice were *s.c.* injected with 2 x 10^5 C7HI cells and 40 days later animals received a daily *i.v.* injection of m-Tyr (n=15, 67 mg/kg) or saline (n=7) for the following 20 consecutive days. At day 60, all treated and control mice were sacrificed and hepatic metastases counted. For LMM3 tumors, BALB/c mice were *s.c.* injected with 2 x 10^5 cells and 25 days later animals received a daily *i.v.* injection of m-Tyr (n=5, 67 mg/kg) or saline (n=5) for the following 20 consecutive days. At day 45, all treated and control mice were sacrificed and metastases counted. *$P<0.05$; *** $P<0.001$ Significant difference.

SUPPLEMENTAL FIGURE 4

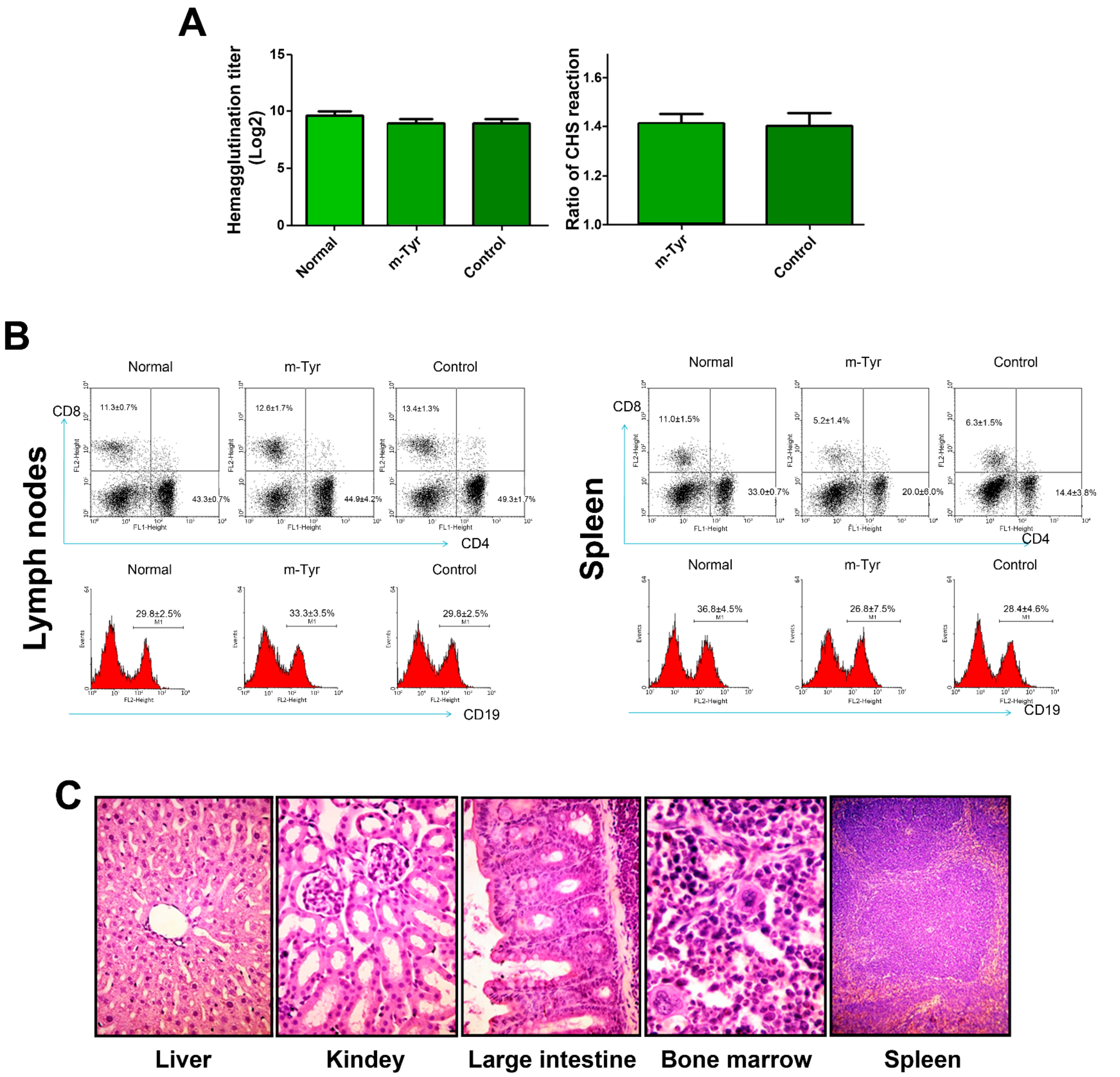

Supplemental Figure 4. **Absence of toxic side effects associated with a periodic administration of m-Tyr.** BALB/c mice received either m-Tyr (67 mg/kg) or saline for 45 consecutive days. A) The hemaglutination titer (humoral immune response) and the ratio of contact hypersensitivity (CHS) reaction (cellular immune response) were quantified and performed as described in supplemental materials. B) Leukocyte subsets in both draining lymph nodes and the spleen were assessed by flow cytometric analysis following m-Tyr treatment with no alterations observed. C) Representative images for H&E staining on liver, kidney, spleen, bone marrow, small and large intestine (25X) following m-Tyr treatment. No significant histological or cytological alterations were observed.
