# Supplementary material for: Game-changing restraint of Ros-damaged phenylalanine, upon tumor metastasis
Source: Cell Death Dis. 2018 Feb 2;9(2):140. doi: 10.1038/s41419-017-0147-8 (PMC5833805; doi:10.1038/s41419-017-0147-8)
Supplement: Supplementary file 2 — supplemental methods for supplemental figures [file 41419_2017_147_MOESM2_ESM.docx]

**Supplemental methods for Supplemental Figures**

**Concomitant resistance experiments for Calu-6 and KB**

Eight to 10-week old male athymic (nu/nu) each weighing at least 20 g, were used in accordance with the “Guidelines for the Welfare of Animals in Experimental Neoplasia” (U.K. Coordinating Committee on Cancer Research).

KB (Human nasopharyngeal carcinoma). Nude mice bearing KB tumors growing subcutaneously (s.c.) in the right flank and measuring 1400mm^3^ (n=6) were challenged with a secondary tumor implant of 1x10^6^ KB tumor cells in the left flank. At the same time, non-tumor bearing nude mice (n=6) received 1x10^6^ KB tumor cells in the left flank and served as control.

Tumors growth in the left flank was periodically monitored, and the inhibition of secondary tumor growth as compared with control, was considered as a measure of concomitant tumor resistance induced by the presence of the primary KB tumor. Experiments were done in triplicates

CALU-6 (Human carcinoma anaplastic of lung). Nude mice bearing CALU-6 tumors growing subcutaneously (s.c.) in the right flank and measuring 1700mm3 (n=6) were challenged with a secondary tumor implant of 2 x 10^6^ CALU-6 tumor cells in the left flank. At the same time, non-tumor bearing nude mice (n=6) received 2 x 10^6^ CALU-6 tumor cells in the left flank and served as control. Tumors growth in the left flank was periodically monitored, and the inhibition of secondary tumor growth as compared with control, was considered as a measure of concomitant tumor resistance induced by the presence of the primary CALU-6 tumor. Experiments were done in triplicates

**Effect of m-Tyr on the the humoral response**.

Control and mTyr-treated mice (*i.v.* administration for 21 days; 67 mg/kg/day) were *i.p.* injected with 1x10^8^ sheep red blood cells (SRBCs). Blood samples were harvested after 7 days, and the titer of SRBC-specific antibodies was determined through a standard hemagglutination assay. m-Tyr treatment did not alter significantly the SRBC-specific humoral immune response.

**Effect of m-Tyr on the cellular immune response.**

Control and mTyr-treated mice (*i.v.* administration for 21 days; 67 mg/kg/day) were subjected to *s.c.* injection with 100 µg of ovoalbumin (OVA) and 7 days later mice were re-challenged with 50 µg of OVA in the right footpad. Footpad size was measured using a caliper prior to the re-challenge with OVA and on the following day; an increase in the right footpad size as compared with the left foot pad or foot pad of controls is indicative of cell influx and the antigen-specific immune response against OVA. m-Tyr treatment did not significantly alter the cellular immune response against OVA.
